# Herbal Medicines (Danggui Liuhuang Decoctions) for Management of Menopausal Symptoms: A Systematic Review of Randomized Controlled Trials

**DOI:** 10.3390/jcm9061778

**Published:** 2020-06-08

**Authors:** Ji Hee Jun, Hye Won Lee, Junhua Zhang, Fengwen Yang, Myeong Soo Lee

**Affiliations:** 1 Clinical Medicine Division, Korea Institute of Oriental Medicine, Daejeon 34054, Korea; zhixi04@kiom.re.kr; 2 Herbal Medicine Research Division, Korea Institute of Oriental Medicine, Daejeon 34054, Korea; hwlee@kiom.re.kr; 3 Evidence Based Medicine Center, Tianjin University of Traditional Chinese Medicine, Tianjin 300193, China; zjhtcm@foxmail.com (J.Z.); 13682027022@163.com (F.Y.)

**Keywords:** Danggui Liuhuang decoction, menopausal symptoms, herbal medicine

## Abstract

Danggui Liuhuang (DLH) decoctions are traditional herbal medicines that are widely used for menopausal symptoms. The objective of this systematic review was to investigate evidence of the efficacy of DLH decoctions for menopausal symptoms. Fifteen databases were searched from inception until 29 May, 2020. We included randomized controlled trials (RCTs) testing any type of DLH decoction. All RCTs investigating DLH decoctions or modified DLH decoctions were included. The methodological quality of the RCTs was evaluated using Cochrane’s risk of bias assessment tool. We measured the certainty of evidence (CoE) according to the GRADE approach. A total of five RCTs met all of the inclusion criteria and were analyzed. The included RCTs had unclear risks of bias in most domains. Based on studies using the Kupperman index for the measurement of menopausal symptoms, DLH decoctions may have ameliorative effects on menopausal symptoms equivalent to those of conventional drug therapies, but we are very uncertain (MD 3.03, 95% CI −3.17 to 9.25, two studies, very low CoE). Compared with conventional drug therapies, DLH decoctions may reduce hot flashes (MD 0.17, 95% CI 0.28 to 0.06, three studies, low CoE). The difference in the response rate between treatments is very uncertain (risk ratio 1.1, 95% CI 1.07 to 1.25, three studies, very low CoE); the results may indicate that compared with drug therapy, DLH decoction therapy elicits responses in 84 more cases per 1000 cases. In conclusion, there is limited evidence that DLH decoctions improve menopausal symptoms equivalently compared with conventional drug therapies. However, the studies had unclear risks of bias, and the CoEs were very low in general. Additional large and rigorous studies are needed.

## 1. Introduction

Menopausal women suffer from a wide variety of symptoms, such as hot flashes, night sweats, depression, anxiety, poor sleep, joint pains, and dry skin, that cause poor quality of life [1,2]. Menopausal women are also vulnerable to cardiovascular disease (CVD) because estrogen withdrawal has a detrimental effect on cardiovascular function and metabolism [3,4]. Hormone replacement therapy (HRT) is the most effective treatment for menopausal syndrome, but current evidence shows that the adverse effects of HRT include increasing the risk of breast cancer. A recent individual participant meta-analysis showed that HRT increases the incidence of breast cancer according to starting age and duration [5]. However, because of the side effects [6,7], many people seek complementary alternative medicines (CAMs) for the management of menopausal symptoms [8,9]. In particular, herbal medicine is the most popular CAM, and a high percentage of menopausal women use it for the management of their symptoms [8,9].

There have been five systematic reviews on the use of various herbal medicines for the management of menopausal symptoms [10,11,12,13,14]. Two reviews concluded that there is insufficient evidence for the effectiveness of herbal medicines in improving menopausal symptoms [12,14]. The third review also reported limited evidence for the ameliorative effects of one herbal product on menopausal symptoms [13]. The fourth review suggested that further study is necessary to confirm the effects of herbal medicine on menopausal hot flashes [10]. The fifth study reported the beneficial effects of adjunctive herbal medicines for menopausal depression [11]. However, these studies assessed the efficacy of various types of herbal medicines together, had intrinsic heterogeneities, and are outdated.

One herbal medicine, Danggui Liuhuang (DLH) decoction, is widely used to treat menopausal symptoms in clinical practice, and its mechanism has been reported in several studies [15,16,17]. However, none of the previous systematic reviews included studies on the use of DLH decoctions for the management of menopausal symptoms. Therefore, the aim of this review was to summarize the results of the randomized controlled trials (RCTs) with the purpose of analyzing the efficacy and safety of DLH decoctions for the treatment of menopausal symptoms.

## 2. Methods

### 2.1. Study Registration and Protocol Information

The protocol of this review was registered with PROSPERO under the number CRD42017079189. We published the protocol in advance and followed the methods to perform this review [18].

### 2.2. Data Source

Fifteen databases were searched from their inception to 29 May, 2020: PubMed, EMBASE, the Cochrane Central Register of Controlled Trials (CENTRAL), AMED, CINAHL, seven Korean medical databases (OASIS, the Korean Traditional Knowledge Portal, the Korean Studies Information Service System, Korea Med, the Korean Medical Database, the Research Information Sharing Service, and DBPia), and three Chinese databases (CNKI, Wanfang, and VIP). We also searched the Chinese clinical trial registry for ongoing trials (http://www.chictr.org.cn/index.aspx). The searches were conducted in Korean, English, and Chinese. The search string used was as follows: “danggui liuhuang decoction” OR “danggui liuhuang tang” OR “danggui liuhuang” and “climacteric” OR “menopause” OR “menopausal” OR “perimenopause” OR “peri-menopausal” OR “perimenopause period” OR “menopausal syndrome” OR “climacteric syndrome” OR “female climacteric syndrome”. The details of the search strategies are in the in the Supplementary Material.

### 2.3. Study Selection

#### 2.3.1. Types of Studies

All RCTs and quasi-RCTs comparing DLH decoctions with Western medicine were included. Case studies, qualitative studies, uncontrolled trials, and reviews were excluded.

#### 2.3.2. Types of Participants

The participants were menopausal women. We excluded women in menopause secondary to surgery, chemotherapy, and/or radiotherapy.

#### 2.3.3. Types of Interventions

Studies using all types of DLH decoctions or modified DLH decoctions were included. The DLH decoctions included the following seven formulas: Angelicae Gigantis Radix, Astragali Radix, Coptidis Rhizoma, Rehmanniae Radix Crudus, Rehmanniae Radix Preparata, Phellodendri Cortex, and Scutellariae Radix. Modified DLH decoctions were defined as single decoctions with one or more supplemental herbs. We excluded DLH decoctions combined with other types of traditional medicine therapies, such as acupuncture, moxibustion, and cupping. The control groups included Western medicine, placebo, and no-treatment groups.

#### 2.3.4. Types of Outcome Measurements

##### Primary Outcomes

Menopausal symptoms (overall, hot flashes, and insomnia) were assessed using response rates and the Kupperman index.

##### Secondary Outcomes

The secondary outcomes included adverse events (AEs).

### 2.4. Data Extraction and Risk of Bias Assessment

#### 2.4.1. Data Extraction

All trials from the electronic database searches were reviewed by two authors (J.H.J. and H.Y.L.). They selected the relevant trials through a review of the titles and abstracts. They extracted data according to the pre-defined criteria. Details such as the participants, interventions, outcomes, and results were obtained from each report.

Any disagreements were resolved by discussion among the two authors (J.H.Z. and H.Y.L.) and an arbiter (M.S.L.). The authors of the included trials were contacted for clarification if necessary. Data were collected from the included trials by two authors (J.H.J. and H.Y.L.).

#### 2.4.2. Risk of Bias

The risk of bias was assessed using the risk of bias assessment tool from the Cochrane Handbook for the following seven domains: (1) random sequence generation, (2) allocation concealment, (3) blinding of participants and personnel, (4) blinding of outcome assessment, (5) incomplete outcome data, (6) selective reporting, and (7) other bias [19]. We used “L”, “H” and “U” as keys for assessment of the risk of bias, with “L” indicating a low risk of bias, “H” indicating a high risk of bias, and “U” indicating an unclear risk of bias. M.S.L. made the final decision as an arbiter for any unresolved disagreements.

#### 2.4.3. Certainty of Evidence

We used the GRADE Pro GDT software (https://gradepro.org/) to create a Summary of Findings table. The certainty of evidence (CoE) was assessed for seven categories: number of studies, study design, risk of bias, inconsistency, indirectness, imprecision, and other considerations [20].

### 2.5. Data Analysis

All of the statistical analyses were performed using Cochrane Collaboration’s software program, Review Manager Software (Version 5.3). For dichotomous data, we present the treatment effects as the relative risk (RR) values with 95% confidence intervals (CIs). Mean differences (MDs) with 95% confidence intervals are used to present the treatment effects for continuous data. We have converted other forms of data into MDs. The Chi-squared test and Higgins I^2^ test were used to assess heterogeneity.

## 3. Results

### 3.1. Description of the Included Trials

**Figure 1:**
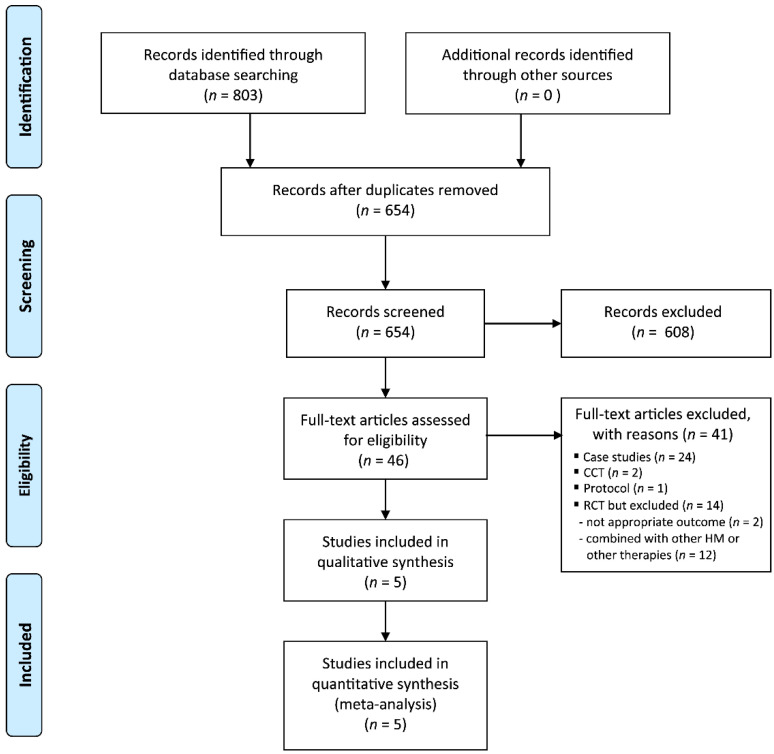
Flow chart of the trial selection process. CCT: clinical controlled trial; RCT: randomized controlled trial.

Our search identified 803 potentially relevant trials, of which five studies were included (Figure 1). The data from all of the included trials are summarized in Table 1 [21,22,23,24,25]. All of the included RCTs were performed in China and were published between 2014 and 2019. Four RCTs measured menopausal symptoms using the Kupperman index [21,22,23,24], and four RCTs assessed response rates [21,23,24,25]. There were no ongoing clinical trials for this topic.

### 3.2. Risk of Bias

**Figure 2:**
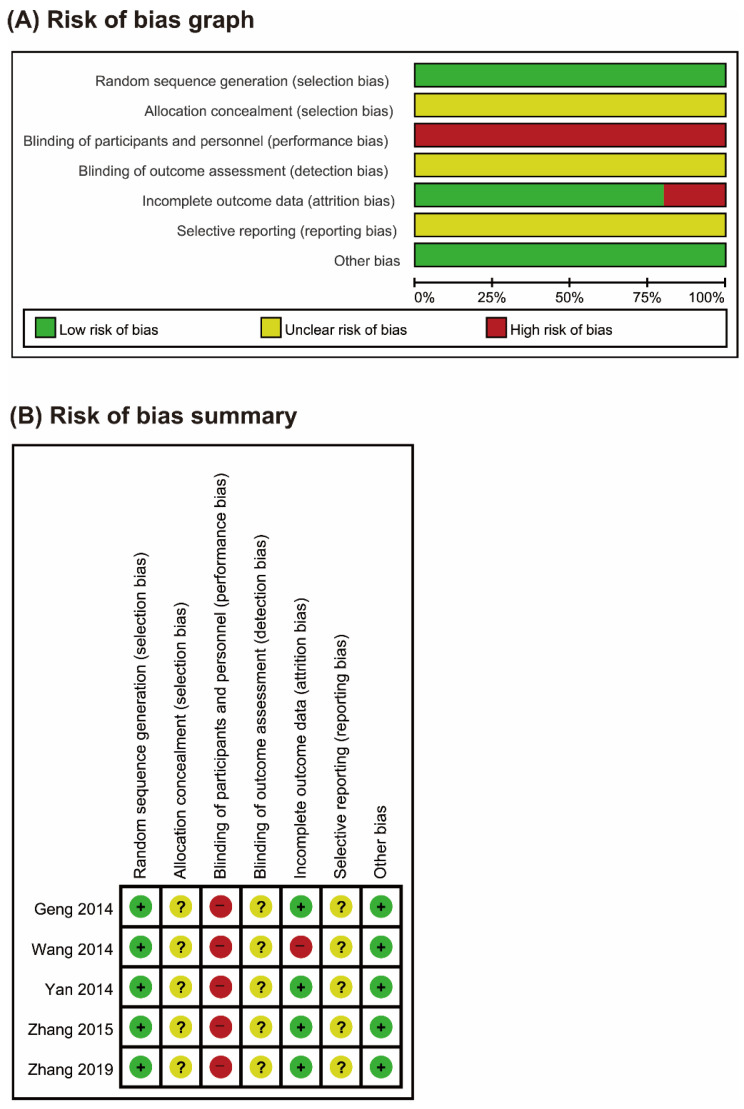
Risks of bias. (**A**) Risks of bias of the included studies. The authors reviewed each item’s risk of bias for each included study. (**B**) Risks of bias of individual studies. +: low risk of bias; −: high risk of bias; ?: unclear.

The risk of bias was unclear in most of the domains for the included RCTs (Figure 2). All of the RCTs reported the use of random sequence generation [21,22,23,24,25], and none of the RCTs mentioned allocation concealment or blinding of outcome assessment. The included trials were comparative studies on DLH decoctions versus conventional drug therapies, and blinding of participants or personnel could not be performed. One study did not report the reasons for drop-out and withdrawal of participants [22]. None of the included studies published or registered their protocols, and the reporting bias was unclear.

### 3.3. Outcome Measurements

#### 3.3.1. Menopausal Symptoms

**Figure 3:**
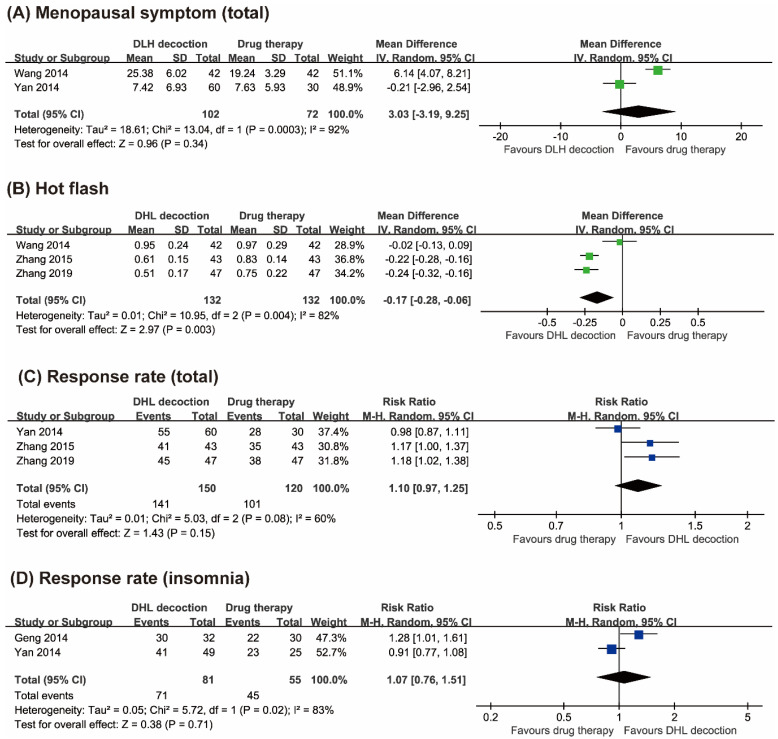
Forest plots of (**A**) Kupperman menopausal index scores, (**B**) hot flash scores, (**C**) response rates (total), and (**D**) response rates (insomnia).

Four RCTs tested the effects of DLH decoctions compared with drug therapies on menopausal symptoms with the Kupperman index [21,22,23,24]. Two studies reported the total menopausal scores [22,23]. The results of the meta-analysis failed to show superior effects of DLH decoctions compared with drug therapies on total menopausal symptom scores (MD 3.30, 95% CI −3.19 to 9.25, two trials, *n* = 174, *p* = 0.34, I^2^ = 92%, Figure 3A).

Three studies reported hot flash scores [21,22,24]. Two studies showed superior effects of DLH decoctions compared with drug therapies in reducing hot flashes [21,24], while the other one failed to do so [22]. The results of the meta-analysis showed favorable effects of DLH decoctions compared with drug therapies in reducing hot flashes (MD −0.17, 95% CI −0.28 to −0.06, three trials, *n* = 264, *p* = 0.003, I^2^ = 82%, Figure 3B).

#### 3.3.2. Response Rate

Three studies assessed response rates based on total menopausal symptoms [21,23,24]. Two RCTs reported superior effects of DLH decoctions compared with drug therapies [21,24], while the other one showed equivalent effects between the two groups [23]. The meta-analysis failed to show superior effects of DLH therapy compared to drug therapy on response rates (RR 1.10, 95% CI 0.97 to 1.25, three trials, *n* = 270, *p* = 0.15, I^2^ = 60%, Figure 3C).

Two RCTs reported the response rates for DLH decoctions compared with drug therapies and showed equivalent effects between the two groups [23,25]. The meta-analysis also failed to show significant differences between the two groups (RR 1.07, 95% CI 0.76 to 1.51, two trials, *n* = 136, *p* = 0.71, I^2^ = 83%, Figure 3D).

#### 3.3.3. Adverse Events

Three RCTs assessed the AEs [22,24,25], while the other two studies did not measure the AEs. One RCT reported several AEs in both groups, including breast pain, gastrointestinal reaction, stomach ache, and vaginal bleeding [22]. The other two studies reported AEs for only drug therapies, including breast pain, vaginal bleeding, daytime sleepiness, dizziness, and fatigue [23,25].

## 4. Discussion

### 4.1. Summary of the Main Results

Few rigorous studies have investigated the effects of DLH decoctions on menopausal symptoms. Evidence from the included studies showed equivalent ameliorative effects between DLH therapy and conventional drug therapies on menopausal symptoms, including hot flashes and insomnia. However, the small number of studies, small sample sizes, and unclear risks of bias prevent firm conclusions from being drawn.

### 4.2. Overall Completeness and Applicability of the Evidence

DLH decoctions may be better than single-agent therapies at improving menopausal symptoms and may reduce both the required doses of conventional drugs and the associated AEs. Despite such promising results, caution should be practiced before generalization of these results, given the diversity of symptoms and the different states of herbal medicines in various countries.

### 4.3. Cernatinty of the Evidence

The CoE was consistently very low or low for all outcomes (Table 2). The most common reasons for the low values were a lack of allocation concealment and a lack of blinding, which are known to result in the overestimation of effect sizes. The CoEs were low due to indirectness and imprecision. High or unclear risks of bias in the included studies reduced their reliability. Although a blinded study was unlikely to be possible due to the use of a decoction, it was unfortunate that no studies provided a placebo decoction to the control group.

### 4.4. Potential Biases in the Review Process

This review has several limitations. Firstly, although considerable efforts were made to retrieve all RCTs on the subject, we cannot be absolutely certain that we were successful. Moreover, selective publishing and reporting are major causes of bias that must be considered. It is conceivable that several RCTs with negative results were not published, which could have distorted the overall picture. Secondly, all of the included studies were conducted in China, and a degree of uncertainty regarding the applications of the findings remains. Finally, the reviewed studies had high risks of bias, and there was a paucity of published studies; therefore, the conclusions of this review might be overstated.

### 4.5. Agreements and Disagreements with Other Studies or Reviews

There have been no previous reviews on this type of herbal decoction. However, the evidence level is similar to that of the systematic reviews on other herbal medicines [10,13,14].

### 4.6. Implications for Practice

The short-term clinical efficacy of the DLH decoctions was good, and their safety was comparable to that of conventional drug therapies. Several approaches were suggested for the management of menopause including lifestyle changes; phytoestrogen; HRT; and sole, combination, or conjugated therapies with estrogen [26]. However, several adverse events associated with HRT and estrogen have been reported, including increased rates of breast cancer. Thus, DLH decoctions appear to be an option for clinical use. However, due to the lack of standardized authoritative assessment indicators of efficacy, the objectivity of the efficacy assessment was compromised. Therefore, we relied mainly on self-reported measures to determine the effects on the main outcomes.

### 4.7. Implications for Research

Two of the main limitations of the included studies were the lack of detailed reporting and the lack of transparency regarding the research process. These limitations resulted in low CoEs and reduced confidence in the pooled results. Future studies should follow the recommended reporting guidelines, including the CONSORT guidelines, to better enable other researchers to understand the study designs and the experimental, analysis, and interpretation methods [27]. They should also utilize adequate allocation concealment, optimal treatment dosages, and sample sizes based on recognized sample size calculations. In addition, important procedures, including the use of validated primary outcome measures and adequate statistical tests for intention-to-treat and missing data, should be undertaken in future research. We excluded the studies with patients with menopause induced by cancer treatment in this study. It may be worthwhile to focus on these patients, who may particularly benefit from nonhormonal treatment, in future study.

In conclusion, the existing studies show equivalent effects of DLH decoctions and conventional drug therapies on menopausal symptoms. However, due to the small number of studies and the high risks of bias, the evidence is limited. Further rigorous RCTs are needed to overcome the many limitations of the current evidence.

## Figures and Tables

**Table 1:** Summary of randomized clinical studies of Danggui Liuhuang (DLH) decoction for menopausal symptoms.

First Author (Year)	Sample Size Conditions (Age)	Intervention Group (Regimen)	Control Group (Regimen)	Main Outcomes	Intergroup Differences	Adverse Event	Composition of Herbal Medicine
Zhang (2019) [21]	94 women who experienced menopausal symptoms (A:50.62; B:50.38)	(A) DLH decoction (2 times daily for 4 weeks, *n* = 47)	(B) Drug therapy (Progynova, 2 mg, once daily for 4 weeks, *n* = 47)	(1) Menopausal symptoms (KI) (2) Response rate	(1) Total: n.r.; hot flash: MD, −0.24 [−0.32, −0.16], *p* < 0.001; insomnia: n.r. (2) Total: RR, 1.18 [1.02, 1.38], *p* = 0.03	n.r.	Angelicae Gigantis Radix 6 g, Astragali Radix 12 g, Coptidis Rhizoma 6 g, Rehmanniae Radix Crudus 12 g, Rehmanniae Radix Preparata 18 g, Phellodendri Cortex 9 g, Scutellariae Radix 9 g
Wang (2014) [22]	168 * women who experienced menopausal symptoms (A: 47.6; B: 48.6)	(A) Modified DLH decoction (2 times daily for 12 weeks, *n* = 42)	(B) Drug therapy (Climen, 1 tablet, once daily for 12 weeks, *n* = 42) (C) TCM patent prescription (*n* = 42) ^†^ (D) A plus C (*n* = 42) ^†^	Menopausal symptoms (KI)	Total: MD, 6.14 [4.07, 8.21], *p* < 0.001; hot flush, MD, −0.02 [−0.13, 0.09], NS.; insomnia: n.r.	Stomachache (A:1, B:3); breast pain (A:11, B: 29); gastrointestinal reaction (B:4); vaginal bleeding (A:2, B:4)	Angelicae Gigantis Radix 15 g, Astragali Radix 30 g, Coptidis Rhizoma 10 g, Rehmanniae Radix Crudus 20 g, Rehmanniae Radix Preparata 20 g, Phellodendri Cortex 15 g, Scutellariae Radix 15 g Hot flushes and sweating (Fossilia Ossis Mastodi 15 g, Ostreae Testa 15 g, Schisandrae Fructus 10 g); Insomnia (Bupleuri Radix 10 g, Zizyphi Semen 20 g); Headaches (Gastrodiae Rhizoma 6 g, Uncariae Ramulus cum Uncus 6 g); Excessive menstrual blood volume (Ecliptae Herba 15 g, Sanguisorbae Radix Carbonisatum 10 g); Emotional disturbance (Tritici Levis Semen 30 g, Glycyrrhizae Radix Preparata 10 g, Zizyphi Fructus 15 g)
Yan (2014) [23]	90 women who experienced menopausal symptoms (n.r.)	(A) Modified DLH decoction (3 times daily or 3 weeks, *n* = 60)	(B) Drug therapy (Tibolone, 1 tablet, 2.5 mg, once daily for 3 weeks, *n* = 30)	(1) Menopausal symptoms (KI) (2) Response rate	(1) Total: MD, −0.21 [−2.96, 2.54], NS.; hot flash: n.r.; insomnia: n.r. (2) Total: RR, 0.98 [0.87, 1.11], NS	n.r.	Angelicae Gigantis Radix 5 g, Astragali Radix 30 g, Coptidis Rhizoma 10 g, Rehmanniae Radix Crudus 20 g, Rehmanniae Radix Preparata 20 g, Phellodendri Cortex 15 g, Scutellariae Radix 15 g, Polygoni Multiflori Caulis 60 g, Zizyphi Semen 20 g
Zhang (2015) [24]	86 women who experienced menopausal symptoms (A: 49.3; B: 48.7)	(A) Modified DLH decoction (2 times daily for 12 weeks, *n* = 43)	(B) Drug therapy (Tibolone tablets, 2.5 mg, once daily for 12 weeks, *n* = 43)	(1) Menopausal symptoms (KI) (2) Response rate	(1) Total: n.r.; hot flash: MD, −0.22 [−0.28, −0.16], *p* < 0.001; insomnia: n.r. (2) Total: RR, 1.17 [1.00, 1.37], *p* < 0.05	Breast pain (B:2); vaginal bleeding (B:2)	Angelicae Gigantis Radix 10 g, Astragali Radix 20 g, Coptidis Rhizoma 3 g, Rehmanniae Radix Crudus 10 g, Rehmanniae Radix Preparata 20 g, Phellodendri Cortex 6 g, Scutellariae Radix 10 g, Nelumbinis Plumula 10 g, Uncariae Ramulus cum Uncus 15 g
Geng (2014) [25]	62 women who experienced menopausal symptoms (insomnia) (A: 50.0; B: 49.5)	(A) Modified DLH decoction (2 times daily for 4 weeks, *n* = 32)	(B) Drug therapy (Estazolam tablets, 1 mg or 3 mg to 4 mg with severe symptoms once daily for 4 weeks, *n* = 30)	Response rate	RR, 1.28 [1.01, 1.61] based on insomnia score	Dizziness (B:7); daytime sleepiness (B:3); fatigue (B:11)	Angelicae Gigantis Radix 15 g, Astragali Radix 30 g, Coptidis Rhizoma 10 g, Rehmanniae Radix Crudus 12 g, Rehmanniae Radix Preparata 10 g, Phellodendri Cortex 12 g, Scutellariae Radix 12 g, Hot flushes and sweating (Lycii Radicis Cortex 15 g); Insomnia (Gardeniae Fructus 9 g, Lophatheri Herba 12 g); Headaches (Chrysanthemi Flos 9 g, Mori Folium 12 g); Emotional disturbance (Lilii Bulbus 15 g); Sweating (Tritici Levis Semen 30 g)

DLH decoction: Danggui Liuhuang decoction; KI: Kupperman index; n.r.: not reported; NS: not significant. * We excluded two additional groups: TCM patent prescription group (*n* = 42) and DLH decoction plus TCM patent prescription group (*n* = 42). ^†^ We excluded these from the analysis because they were not in the included criteria.

**Table 2:** Summary of findings.

DLH Decoction Compared to Conventional Drug Therapies for Menopausal Symptoms					
Patient or Population: Menopausal Symptoms Setting: community/ outpatients Intervention: DLH Decoction Comparison: Conventional Drug Therapies					
Outcomes	No. of Participants (Studies) Follow-up	Certainty of the Evidence (GRADE)	Relative Effect (95% CI)	Anticipated Absolute Effects^*^	
				Risk with Conventional Drug Therapies	Risk Difference with DLH Decoction
Menopausal symptoms Kupperman index (follow up: range 3 weeks to 12 weeks)	174 (2 RCTs)	⨁◯◯◯ VERY LOW ^a,b,c,d^	-		MD 3.03 higher (3.19 lower to 9.25 higher)
Hot flashes (follow up: range 3 weeks to 12 weeks)	264 (3 RCTs)	⨁⨁◯◯ LOW ^a,d^	-		MD 0.17 lower (0.28 lower to 0.06 lower)
Response rate (follow up: range 3 weeks to 12 weeks)	270 (3 RCTs)	⨁◯◯◯ VERY LOW ^a,b,d^	RR 1.10 (1.07 to 1.25)	842 per 1000	84 more per 1000 (59 more to 210 more)
Response rate (insomnia) (follow up: range 3 weeks to 12 weeks)	136 (2 RCTs)	⨁◯◯◯ VERY LOW ^a,b,d^	RR 1.07 (0.76 to 1.51)	818 per 1000	57 more per 1000 (196 fewer to 417 more)

* The risk in the intervention group (and its 95% confidence interval) is based on the assumed risk in the comparison group and the relative effect of the intervention (and its 95% CI). CI: Confidence interval; DLH: Danggui Liuhuang; MD: Mean difference; RR: Risk ratio. GRADE Working Group grades of evidence: High certainty (⨁⨁⨁⨁): We are very confident that the true effect lies close to that of the estimate of the effect. Moderate certainty (⨁⨁⨁◯): We are moderately confident in the effect estimate: The true effect is likely to be close to the estimate of the effect, but there is a possibility that it is substantially different. Low certainty (⨁⨁◯◯): Our confidence in the effect estimate is limited: The true effect may be substantially different from the estimate of the effect. Very low certainty (⨁◯◯◯): We have very little confidence in the effect estimate: The true effect is likely to be substantially different from the estimate of effect. Explanations: ^a^ Downgraded by one level for study limitations: unclear or high risk of bias in half of the domains in included studies. ^b^ Downgraded by one level for substantial heterogeneity in included studies. ^c^ Downgraded by two levels for imprecision: wide confidence interval crossing assumed threshold of minimal clinically important difference. ^d^ Downgraded by one level for imprecision: small sample size.

## Supplementary Materials

The following are available online at https://www.mdpi.com/2077-0383/9/6/1778/s1, supplementary material: Search strategies.
