# Supplementary material for: Herbal Medicines (Danggui Liuhuang Decoctions) for Management of Menopausal Symptoms: A Systematic Review of Randomized Controlled Trials
Source: J Clin Med. 2020 Jun 8;9(6):1778. doi: 10.3390/jcm9061778 (PMC7355969; doi:10.3390/jcm9061778)
Supplement: Supplementary file 1 [file jcm-09-01778-s001.pdf]

Supplement 1. Search strategies

1. CNKI, Wanfang, VIP

#1. 更年期

#2. 绝经期

#3. 更年期综合征

#4. 更年期症状

#5. 围绝经期

#6. climacteric

#7. menopause

#8. menopausal

#9. perimenopause

#10. peri-menopausal

#11. perimenopause period

#12. menopausal syndrome

#13. climacteric syndrome

#14. Female Climacteric Syndrome

#15. OR /1-14

#16. 当归六黄

#17. 当归六黄汤

#18. Dagngui liuhuang decoction

#19. Danggui liuhuang tang

#20. Danggui liuhuang

#21. OR/16-20

#22. 15 AND 21

2. PubMed

#1.Menopause [MeSH]

#2. Menopause, premature [MeSH]

#3. Postmenopause [MeSH]
#4. Climacteric [MeSH]
#5. menopause* OR perimenopaus* OR peri-menopaus* OR post menopause* OR post-menopaus OR climact* [tw]
#6. Climacteric symptoms [tw]
#7. OR/1-6
#8. "Medicine, Chinese Traditional" [Mesh]
#9. "Medicine, Kampo"[Mesh]
#10. "Medicine, Korean Traditional"[Mesh]
#11. "Dangguiliuhuang"
#12. "Danggui liughuang decoction"
#13. "Danggui liuhuang tang"
#14. OR/8-13
#15. #7 AND #14

3. CENTRAL

#1. Menopause [MeSH]

#2. Menopause, premature [MeSH]

#3. Postmenopause [MeSH]

#5. Climacteric [MeSH]

#6. Menopause OR perimenopause OR postmenopause OR climacteric [TIAB]

#7. OR/ 1-6

#8. "Medicine, Chinese Traditional" [Mesh]

#9. "Medicine, Kampo"[Mesh]

#10. "Medicine, Korean Traditional"[Mesh]

#11. "Dangguiliuhuang"

#12. "Danggui liughuang decoction"

#13. "Danggui liuhuang tang"

#14, OR/ 8-13

#14. #7 AND #14

4. EMBASE

#1. exp climacterium

#2. exp menopause/ or exp menopause related disorder/ or exp "menopause and climacterium"

#3. exp early menopause

#4. exp postmenopause

#5. (menopaus$ or perimenopaus$ or postmenopaus$).tw.

#6. climacter$.tw.

#7. OR/1-6

#8. “Chines medicine”[Mesh]

#9. Kampo medicine [Mesh]

#10. Korean medicine .tw.

#11. Traditional Korean medicine.tw.

#12. “Dangguiliuhuang”.tw.

#13. “Danggui liughuang decoction” .tw.

#14. “Danggui liuhuang tang” .tw.

#15. OR/8-14

#15. #7 AND #15

5. CINAHL

Danggui liuhuang OR Herbal medicine AND Menopause OR perimenopause OR postmenopause OR climacteric

6. OASIS, Korea Traditional Knowledge Portal, Korea studies information service system, DBpia

당귀육황탕 OR 한약 OR Herbal AND 갱년기 OR 폐경 OR climacterics

7. Korea Med

("Herbal Medicine" [MH] or "Phytotherapy" [MH] or "Drugs, Chinese Herbal" [MH] or Herb*[tw] or Phytotherap*[tw]) and ("Menopause" [MH] or "Perimenopause" [MH] or "Postmenopause" [MH] or Menopaus*[tw] or perimenopaus*[tw] or postmenopaus*[tw] or "Climacteric" [MH] or "Hot Flashes" [MH] or Climacteric[tw] or "hot flash*"[tw] or "hot flush*"[tw])

8. Korean Medical Databases

Danggui liuhuang OR Herbal medicine AND Menopause OR perimenopause OR postmenopause OR climacteric
